# Ultrastructural Analysis of *Leishmania infantum chagasi* Promastigotes Forms Treated *In Vitro* with Usnic Acid

**DOI:** 10.1155/2015/617401

**Published:** 2015-02-12

**Authors:** João S. B. da Luz, Erwelly B. de Oliveira, Monica C. B. Martins, Nicácio H. da Silva, Luiz C. Alves, Fábio A. B. dos Santos, Luiz L. S. da Silva, Eliete C. Silva, Paloma L. de Medeiros

**Affiliations:** ^1^Pos-Graduation Program in Pathology, Federal University of Pernambuco, Avenida Prof. Moraes Rego 1235, 50670-901 Recife, PE, Brazil; ^2^Pos-Graduation Program in Therapeutic Innovation, Federal University of Pernambuco, Avenida Prof. Moraes Rego 1235, 50670-901 Recife, PE, Brazil; ^3^Department of Histology and Embryology, Federal University of Pernambuco, Avenida Prof. Moraes Rego 1235, 50670-901 Recife, PE, Brazil; ^4^Department of Biochemistry, Federal University of Pernambuco, Avenida Prof. Moraes Rego 1235, 50670-901 Recife, PE, Brazil; ^5^Laboratory of Immunopathology Keizo Asami (LIKA) and Research Center Aggeu Magalhães, CPqAM/FIOCRUZ, Federal University of Pernambuco, Avenida Prof. Moraes Rego, 1235, 50670-901 Recife, PE, Brazil

## Abstract

Leishmaniasis is considered by the World Health Organization as one of the infectious parasitic diseases endemic of great relevance and a global public health problem. Pentavalent antimonials used for treatment of this disease are limited and new phytochemicals emerge as an alternative to existing treatments, due to the low toxicity and cost reduction. Usnic acid is uniquely found in lichens and is especially abundant in genera such as* Alectoria*,* Cladonia*,* Evernia*,* Lecanora*,* Ramalina,* and* Usnea*. Usnic acid has been shown to exhibit antiviral, antiprotozoal, antiproliferative, anti-inflammatory, and analgesic activity. The aim of this study was to evaluate the antileishmanial activity of usnic acid on* Leishmania infantum chagasi* promastigotes and the occurrence of drug-induced ultrastructural damage in the parasite. Usnic acid was effective against the promastigote forms (IC_50_ = 18.30 ± 2.00 *µ*g/mL). Structural and ultrastructural aspects of parasite were analyzed. Morphological alterations were observed as blebs in cell membrane and shapes given off, increasing the number of cytoplasmic vacuoles, and cellular and mitochondrial swelling, with loss of cell polarity. We concluded that the usnic acid presented antileishmanial activity against promastigote forms of* Leishmania infantum chagasi* and structural and ultrastructural analysis reinforces its cytotoxicity. Further,* in vitro* studies are warranted to further evaluate this potential.

## 1. Introduction

Leishmaniasis is one of the most neglected diseases that remain a public health problem worldwide, affecting approximately 12 million people in more than 98 countries, with more than 350 million people at risk and when not treated, death is expected to occur after a period of four months to one year [1, 2].

Leishmaniasis is endemic in areas of tropics and subtropics, including southern Europe, Asia, Africa, and the Americas [3–5].

*Leishmania infantum chagasi*, a trypanosomatid parasite, is an etiological agent of visceral leishmaniasis (VL) in the American continent and it is now admitted to be the same species causing visceral leishmaniasis in Europe and certain parts of Africa [6].

Pentavalent antimonials such as sodium stibogluconate and meglumine antimoniate have been used in the treatment of all forms of leishmaniasis for more than half a century; although the mechanism of action of pentavalent antimonial is not fully understood, it is generally accepted that the active form of the metal is the reduced form Sb (III), which can lead to serious limitations due to high toxicity and resistance to drugs [2, 7]. In addition, a major problem in antimonial chemotherapy is the emergence of clinical resistance against pentavalent antimonial drugs that has reached epidemic proportions in parts of India [8–10]. Newer drugs such as miltefosine and amphotericin B have shown promising results, but they also cause some side effects that may limit their use. In this context, the discovery of new active and promising compounds with antileishmanial potential remains essential for control and prevention of leishmaniasis [11, 12].

Several natural products with antileishmanial activity have been recently reported, including naphthoquinones, lignans, neolignans, alkaloids, flavanol, and terpenoids [13, 14], but none has reached clinical use.

Lichens synthesize over eight hundred types of metabolites [15] and depsides, depsidones, dibenzofurans, xanthenes, anthraquinones, and usnic acids are amongst the more extensively studied lichen metabolites [16]. The search for new phytochemicals with antiparasitic action emerges as an alternative to existing treatments, due to the low toxicity and cost reduction.

Some studies showed that usnic acid, secondary metabolite of the lichen* Cladonia substellata* Vainio, was initially used in the treatment of pulmonary tuberculosis [17], and there are data regarding its biological activities as antibiotic [18], antiproliferative [19], analgesic and antipyretic [20], anti-inflammatory [21], antiviral [22, 23], antifungal [24], against the parasite* Trypanosoma cruzi* [25], and an immunologic modulator [26]. In addition, its mutagenic and cytotoxic activities have been determined against normal and malignant human cells lines [27–30].

Taking into account the side effects and the resistance that pathogenic protozoan parasites develop against drugs currently used in the treatment of leishmaniasis, more attention should be given to extracts and biologically active compounds isolated from plant species commonly used in herbal medicine. Thus, in this work, we have analyzed the* in vitro *effect of usnic acid on growth and ultrastructure of* Leishmania infantum chagasi* promastigote forms.

## 2. Materials and Methods

### 2.1. Extraction of Usnic Acid

Usnic acid was isolated from the crude extract of the lichen Cladonia substellata Vainio, and was obtained from Dr. Nicácio Henrique da Silva at Department of Biochemistry, Federal University of Pernambuco. Fractionation and purification of this compound were performed as previously described [31]. A stock solution was prepared at 10 mg/mL in DMSO and stored at 4°C until use.

### 2.2. Parasites Culture

The strain of* Leishmania infantum chagasi* (MHOM/BR2000/Merivaldo2) was kindly provided by Dr. Osvaldo Pompílio de Melo Neto at Department of Microbiology of Research Center Aggeu Magalhães, FIOCRUZ. The promastigotes were routinely grown in Liver Infusion Tryptose medium (LIT) at 26°C, supplemented with 10% heat-inactivated fetal bovine serum (FBS) (LGC Biotechnology), 0.1% penicillin and streptomycin, and 0.2% hemin (Sigma).

### 2.3. *In Vitro* Antileishmanial Activity

The antileishmanial activity was assessed by the colorimetric method MTT [3-{4.5-dimethylthiazol-2-yl}-2.5-diphenyl-tetrazolium] (SIGMA) that is based on the conversion of the tetrazolium salt into the colored formazan product, the concentration of which can be determined spectrophotometrically. The promastigotes were seeded (2 × 10^6^ cells/mL) with LIT medium in 96-well microplates and incubated with 50 *μ*L of different concentrations of usnic acid (100 to 0.195 *μ*g/mL) and amphotericin B (positive control). LIT medium (50 *μ*L) (negative control) was also added. After 72 h of incubation with usnic acid and amphotericin B 25 *μ*L of MTT solution was added (5 mg/mL). After 3 h of incubation at 37°C, MTT solution was aspirated and 100 *μ*L DMSO was added for the solubilization of the formazan crystals. After solubilization, the absorbance was determined in a spectrophotometer at 585 nm wavelength. The results were expressed as percentage of relative viability of cells to the negative control group [32]. All the experiments were performed in triplicate.

### 2.4. Scanning Electron Microscopy

To evaluate parasite ultrastructural alterations by scanning electron microscopy,* Leishmania infantum chagasi* promastigotes were grown for 72 h as described in LIT-DMSO or the same medium containing 25 *μ*g/mL usnic acid; they were subsequently collected by centrifugation at 1500 g, washed twice in 0.1 M Phosphate buffer (pH 7.2), and fixed in a solution containing 2.5% glutaraldehyde, 4% paraformaldehyde, and 0.1 M T phosphate buffer. After washing twice in the same buffer, the parasites were adhered to glass slides previously coated with 0.1% poly-I-lysine for 30 min at 37°C. Subsequently, the slides were washed twice with 0.1 M phosphate buffer, postfixed in solution of OsO_4_ for 1 h at room temperature, and washed twice again with 0.1 M phosphate buffer. All samples were dehydrated gradually increasing the ethanol concentrations (30–100%) and were critical point dried using CO_2_, mounted on metal stubs, and coated with gold (5–30 nm) for observation in a scanning electron microscope (JEOL T-200).

### 2.5. Transmission Electron Microscopy

Control and treated culture promastigotes with 25 *μ*g/mL were harvested, at room temperature by centrifugation at 1500 g, washed twice in PBS, and fixed for 2 h at 4°C in a solution containing 2.5% glutaraldehyde, 4% paraformaldehyde, and 0.1 M cacodylate buffer, pH 7.2. After washing in this similar buffer, cells were postfixed for 1 h in a solution containing 1% OsO_4_, 0.8% potassium ferricyanide, and 5 mM CaCl_2_ in 0.1 M cacodylate buffer, pH 7.2. The cells were then dehydrated in acetone and embedded in epoxy resin. Ultrathin sections were stained with uranyl acetate and lead citrate and examined in a Zeiss EM 109 transmission electron microscope.

### 2.6. Statistical Analysis

Statistical analysis of growth differences between treated and control cultures (IC_50_) was performed using the Anova with Tukey test, with *P* < 0.05. The results were expressed as mean values ± standard deviation (S.D.).

## 3. Results

**Figure 1:**
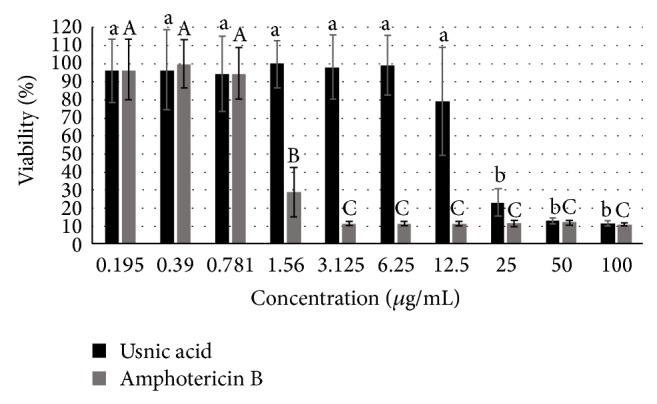
*In vitro* effects of different concentrations of usnic acid and amphotericin B on the growth of* Leishmania infantum chagasi* promastigote forms. Means followed by different letters indicating statistically significant differences according to the Anova-Tukey test (*P* < 0.05, compared to controls).

The antileishmanial activity was directly proportional to usnic acid and amphotericin B concentrations. Usnic acid antileishmanial activity was estimated by the IC_50_ concentration at 72 h after incubation. Usnic acid was found to exhibit a lower inhibitory activity against* Leishmania infantum chagasi* (IC_50_ = 18.30 ± 2.00 *μ*g/mL) than the reference drug amphotericin B (1.36 ± 0.067 *μ*g/mL) (Figure 1).

**Figure 2:**
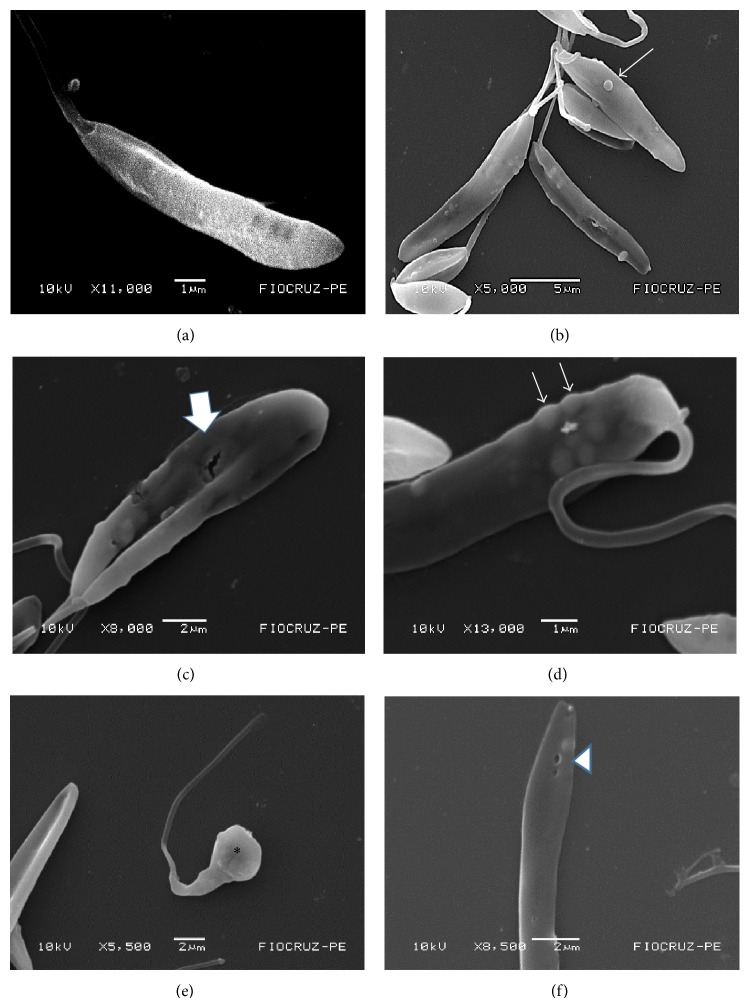
Scanning electron micrographs of* Leishmania infantum chagasi* promastigotes exposed to usnic acid. (a) Control group (no treatment); (b, c, d, e, and f) treated group usnic acid at a concentration of 25 *μ*g/mL for 72 hours. Blebs on the surface of promastigotes (thin arrows), membrane rupture (full arrow), swelling (asterisk), and the formation pore (arrowhead). Bars: 1 *μ*m (a, d); 2 *μ*m (c, e and f). Magnifications: (a) 11.000x, (b) 5.000x, (c) 8.000x, (d) 13.000x, (e) 5.500x, and (f) 8.500x.

The analysis of scanning electron micrographs of treated parasites demonstrated that usnic acid affected the parasite surface. Morphological alterations were observed as the presence of blebs in the cell membrane and of shapes given off. No ultrastructural change was observed in promastigotes grown with LIT-DMSO for 72 h, showing the elongated normal morphology (Figures 2(a)–2(f)).

**Figure 3:**
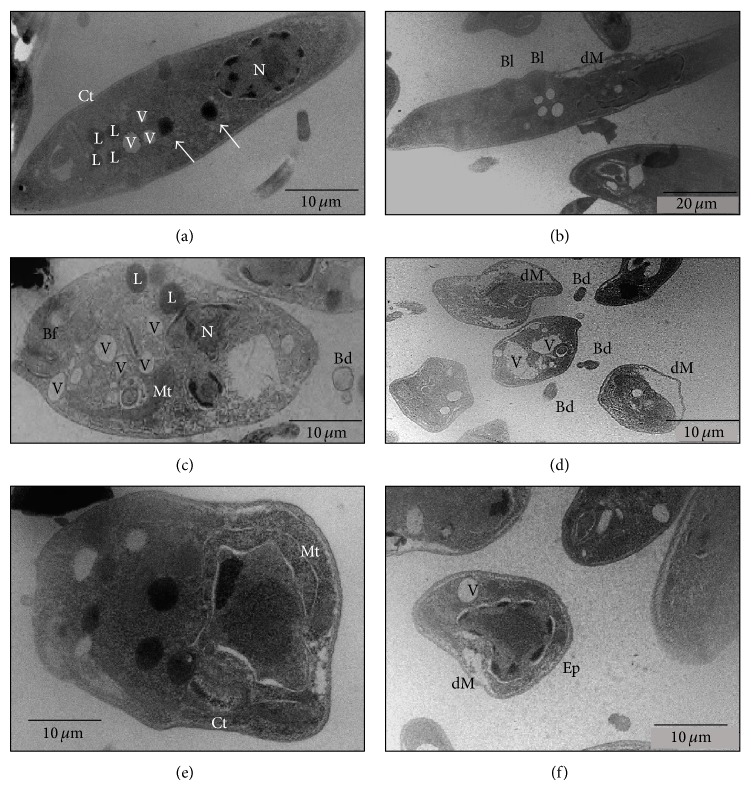
Transmission electron micrographs promastigote forms of* Leishmania infantum chagasi*. (a) Control group; (b), (c), (d), (e), and (f) treated groups (AU) at a concentration of 25 *μ*g/mL for 72 hours. Vacuoles (V), acidocalcisomes (arrows), nucleus (N), lipid (L), kinetoplast (Ct), flagellar pocket (Bf), mitochondrial swelling (Mt), detachment of the plasma membrane (dM), blebs (Bl), detached blebs (Bd), and electron-lucent space (Ep). Bars 10 *μ*m (a, c, d, e, and f), Bars 20 *μ*m (b).

Morphological alterations of Leishmania infantum chagasi promastigotes treated with 25 *μ*g/mL usnic acid were observed by transmission electron microscopy. The compound caused changes in the cytoplasm density, cell swelling, and loss of cell polarity. Changes in mitochondrial morphology were characterized by marked swelling. We also noticed an increased number of intracellular vacuoles. Blebs were observed in the plasma membrane and detached from the membrane. It was also observed that the membrane was separated from the cytoplasm and there was a higher accumulation of fat compared to controls. Nucleus was observed with presence of an electron-lucent space, when compared to the control (Figures 3(a)–3(f)).

## 4. Discussion

Usnic acid is the most abundant constituent of various species of lichens, including those belonging to the genus* Cladonia and Usnea* [33]. This compound is known to present several biological activities, acting as an antiprotozoan [34, 35] and antiparasitic [25]. Research with usnic acid* in vitro* against promastigote forms of* Leishmania *sp. demonstrated the effectiveness of usnic acid in their antileishmanial activity [35].

The concentration which inhibited 50% growth of a culture of* Leishmania infantum chagasi* promastigote forms obtained in our experiments (18.30 *μ*g/mL) was close to that obtained by de Carvalho et al. [25] who detected an IC_50_ around 20 *μ*g/mL for another trypanosomatid parasite (*T. cruzi*). Our results demonstrated a promising leishmanicidal activity of usnic acid against the* Leishmania infantum chagasi* promastigote forms* in vitro* when confronted with the study of Jota et al. [36] who obtained for other depsides from lichens (atranorin and norstictic acid), IC_50_ values for* Leishmania infantum chagasi* at concentrations of 30 and 40 *µ*g/mL, respectively.

In a literature review conducted until October 2006 few references regarding the leishmanicidal activity of compounds isolated from lichens were found. Fournet et al. [35] revealed that among the lichen substances tested* in vitro* against promastigote forms of* L. braziliensis*,* L. amazonensis* and* L. donovani*, the usnic acid presented total lysis of parasites in the concentration of 25 *µ*g/mL. The promastigote forms of* L. amazonensis* and* L. braziliensis* under the effect of usnic acid exhibited lysis of parasites at concentration of 10 *µ*g/mL (80–90%) and against* L. donovani* the activity was total. Already, depsidones (panarin and 1-cloropanarin) were active only in concentrations equal to or greater than 50 *μ*g/mL.

Scanning electron micrographs reveal the formation of blebs in the plasma membrane, membrane rupture, and loss of polarity. da Silva et al. [6] when using warifteine drug against promastigotes of* Leishmania infantum chagasi* found similar results in scanning microscopy, such as blebs, suggesting that usnic acid metabolism operates through a change in intracellular calcium concentration.

Comparing the ultrastructural effects of usnic acid with those produced by other drug (amiodarone), against the promastigotes and amastigotes forms of* L. amazonensis* evaluated by da Silva et al. [6], was observed similar effects on the membranes of parasites, such as lipid accumulation and loss of integrity of nuclear envelope and plasma membrane, leading to cell death by necrosis.

According to Abo-Khatwa et al. [37] usnic acid would be similar to the classical uncoupler 2, 4-dinitrophenol (DNP) oxidative phosphorylation, and thereby promote ATP depletion. Furthermore, usnic acid has lipophilic characteristics and ionizing could alter the mitochondrial membrane, causing damage of this organelle [15].

Einarsdóttir et al. [38] showed that mitochondrial membrane potential loss appears to be dose-dependent with respect to the effect of usnic acid in the two cell lines such as breast cancer T-47D and pancreatic cancer Capan-2. These data demonstrate and confirm one of the mechanisms of action of usnic acid previously cited by Müller [15], where changes in membrane lipid composition alter the physical properties with loss of mitochondrial function. We suggest, based on prior observations, that the usnic acid destabilized membranes allowing the leishmanicidal effect.

Adade and Souto-Padrón [39] studying the ultrastructures of the genera* Trypanosoma* and* Leishmania* regarding the antiparasitic drugs observed that the ultrastructural effects are similar among the various drugs used as well as between herbal medicines, including usnic acid.

The study in question was relevant to effectiveness of usnic acid in the face of* Leishmania infantum chagasi *promastigote forms, which leads us to suggest it as a possible phytotherapic agent in the treatment of visceral leishmaniasis. In this context, further studies are needed to elucidate the mechanisms of action of usnic acid and its physiological effects* in vivo.*
